# Predictive model to identify multiple synergistic effects of geriatric syndromes on quality of life in older adults: a hospital-based pilot study

**DOI:** 10.1186/s12877-025-05931-8

**Published:** 2025-04-26

**Authors:** Chien-Chou Su, Yung-Chen Yu, Deng-Chi Yang

**Affiliations:** 1 Clinical Innovation and Research Center, National Cheng Kung University Hospital, College of Medicine, National Cheng Kung University, Tainan, Taiwan; 2 Department of Nursing, National Cheng Kung University Hospital, College of Medicine, National Cheng Kung University, Tainan, Taiwan; 3 Department of Geriatrics and Gerontology, National Cheng Kung University Hospital, College of Medicine, National Cheng Kung University, No. 138 Sheng Li Road, Tainan City, 704 Tainan, Taiwan; 4 School of Medicine, College of Medicine, National Cheng Kung University, Tainan, Taiwan; 5 Institute of Clinical Pharmacy and Pharmaceutical Sciences, College of Medicine, National Cheng Kung University, Tainan, Taiwan

**Keywords:** Older adults, Quality of life, Geriatric syndromes, Machine learning

## Abstract

**Background:**

Quality of life (QOL) has been reported to be associated with sociodemographic characteristics and geriatric syndromes in older adults, but the impact of interactions among multiple geriatric syndromes on QOL remains unexplored. We aimed to apply a machine learning method to evaluate the effects of interactions among multiple geriatric syndromes on QOL in older adults.

**Methods:**

We recruited adults aged ≥ 65 years admitted to a tertiary medical center from June 2018 to September 2018. The main outcome was the three-level five-dimensional Euro-Quality of Life tool (EQ-5D-3 L) utility value. The random forest algorithm was used to identify and rank the strongest predictors of geriatric syndromes. The relation between predictors and outcomes was visualized with accumulated local effects plots and interaction effects. Model performance was evaluated by 5-fold cross-validation with metrics of R-square, the mean square error of estimation and the mean absolute error of estimation.

**Results:**

The study included 160 older adults with a mean age of 79 years. The top ten features that significantly influenced the utility prediction were activities of daily living (ADL), frailty, pain, the number of medications used, age, depression, the Charlson Comorbidity Index (CCI), body mass index (BMI), peptic ulcer, and emotional loneliness. The two-way interactions between ADL, frailty, and pain significantly interacted with other predictors.

**Conclusion:**

ADL, frailty, and pain are important factors to be considered when assessing QOL in older adults. It is important for clinicians to consider them together in clinical decision-making.

**Supplementary Information:**

The online version contains supplementary material available at 10.1186/s12877-025-05931-8.

## Introduction

With the aging of the population and advances in medicine, the goal of health outcomes has shifted from morbidity and mortality to well-being and quality of life (QOL). It is a multidimensional construct complexly influenced by an individual’s physical health, psychological state, level of independence, social relationships, and environmental interactions [1].

Better QOL has been reported to be associated with higher education level [2, 3], higher income [2, 4], being engaged in social activities [2, 3], living with spouse [3], and having social support [4]. Poorer QOL has been reported to be associated with older age [2, 4], female gender [2, 5], social isolation [2, 6], and multiple morbidities [2, 7]. In addition, previous studies have indicated that poor QOL is associated with the presence of geriatric syndromes, including cognitive impairment [6, 8], functional impairment [3, 6–8], depression [4, 6, 8], falls [7], and polypharmacy [2].

However, the interactions among multiple geriatric syndromes on QOL remain unexplored. According to previous studies, multiple factors influence QOL in older adults, and these factors may exhibit nonlinearity and interact with each other [9]. It is difficult to use a traditional linear model to investigate the association between effects of interactions among multiple geriatric syndromes and QOL [10]. Traditional linear regression requires a priori identification of predictor interactions, a process that becomes increasingly complex and unreliable as the number of predictors grows. This approach is vulnerable to multicollinearity, particularly in high-dimensional spaces, potentially leading to biased estimates when interaction terms are incorporated. In contrast, machine learning algorithms are well-suited for handling high-dimensional, nonlinear data with multiple interactions, thereby overcoming the inherent limitations of classical linear regression techniques [11]. The objective of the present study was to apply a machine learning approach to assess the interactions among multiple geriatric syndromes on QOL in older adults.

## Materials and methods

### Participants

We recruited individuals aged ≥ 65 years admitted to medical wards at a tertiary medical center from June to September 2018. Individuals with hospital stay < 72 h, delirium, critical or terminal conditions, impaired communication abilities, or long-term bedridden were excluded. Impaired communication abilities were defined as the inability of older adults to respond to our questionnaire due to severe cognitive impairment, or severe hearing impairment even with the use of hearing aids. We excluded older adults with acute delirium or impaired communication abilities because they may not respond to our questionnaire correctly. We excluded older adults with long-term bedridden because those with long-term bed-ridden were associated with poor quality of life [12]. This investigation was conducted in strict accordance with established ethical guidelines, and informed consent was procured from all study participants following a comprehensive disclosure of the research protocol. Approval for the study was granted by the Institutional Review Board of National Cheng Kung University Hospital (A-ER-106-261).

Upon enrollment, eligible participants underwent a face-to-face comprehensive structural questionnaire interview within 72 h after their admission. The collected data included pertinent participant information, including demographic characteristics, comorbidities, and various geriatric syndromes.

### Demographic characteristics

We collected data on the participants’ demographic attributes, including their age, sex, body mass index (BMI), marital status, family support, educational attainment, household income, and institutionalization status. Marital status was further stratified into two categories: married or living with a partner and living alone due to separation, divorce, widowhood, or never married [13]. Educational level was classified into three distinct categories: illiterate, elementary school, and high school or above.

### Comorbidity

To collect data on comorbid diseases, we conducted a review of the electronic medical records and determined the Charlson Comorbidity Index (CCI) [14]. As a participant’s CCI increases, they face a higher risk of mortality within the next decade [15]. In the current study, we used the CCI to show the comorbidity status.

### Comprehensive geriatric assessment (CGA)

CGA was first developed in the United Kingdom, and its concepts, indications, and applications evolved over time [16]. It is a multidomain, multidisciplinary diagnostic and therapeutic process performed to assess the physical, mental, social, and functional domains of older adults [17]. The physical domain includes the number of drugs used, nutrition status, urinary incontinence, and falls; the mental domain includes cognition and mood; the social domain includes living condition, marital status, and educational level; and the functional domain includes a frailty assessment. All the components of the CGA mentioned above were completed within 72 h after admission. For the physical domain, the number of medications used was reported by the individuals themselves. The assessment of activities of daily living was conducted using the Minimum Data Set-Activities of Daily Living (MDS-ADL) scale [18], with higher scores indicating a greater degree of dependence. Pain was defined by asking the individuals if they had pain or not, and it was confirmed if the answer was yes. Nutrition status was assessed by the Malnutrition Universal Screening Tool (MUST) [19]. It is a screening tool used to categorize adults into low-risk (MUST score = 0), medium-risk (MUST score = 1), and high-risk categories for malnutrition (MUST score ≥ 2) [19]. The participants with MUST scores ≥ 2 were defined as having malnutrition in the current study. Urinary incontinence was defined if the individuals reported leakage of urine on $\:\ge\:$ 6 days within the past year. Fall was defined according to 1987 the Kellogg International Working Group on the prevention of falls in the older adults as ‘unintentionally coming to the ground or some lower level and other than as a consequence of sustaining a violent blow, loss of consciousness, sudden onset of paralysis as in stroke or an epileptic seizure‘ [20]. A fall was indicated if fall episodes occurred ≥ 2 times in the past 6 months before admission. For the mental domain, cognition was measured by the Short Portable Mental Status Questionnaire, which is a 10-item screening tool that was developed in 1975 [21]. Incorrect answers on more than 2 questions indicated impaired cognitive function. One more incorrect answer was allowed for the participants with a grade school education or lower, and one less incorrect answer was allowed for the participants with a high school education or higher [21]. Mood state was evaluated by the geriatric depression scale-5 (GDS-5), which is a 5-item screening tool that was developed in 1999 [22]. The GDS scores ranged from 0–5, with a sensitivity of 0.97 and a specificity of 0.85 using a score ≥ 2 as a cutoff point to define the presence of depressive symptoms [22]. For the functional domain, frailty was assessed by the clinical frailty scale (CFS) [23]. The CFS is a simple and intuitive clinical examination with a 9-point scale, and older adults with CFS ≥ 4 were classified as frail within 72 hours after admission.

### Quality of life and utility value

QOL was assessed with the three-level five-dimensional Euro-Quality of Life tool (EQ-5D-3 L) [24]. It was selected for its widespread use and ease of administration. It demonstrates satisfactory discriminatory power with respect to construct properties, and is a feasible and valid measure in the older population [25]. The EQ-5D contains five dimensions, including mobility, self-care, usual activities, pain/discomfort, and anxiety/depression. The assessment of each dimension involved categorization into three levels: no problems, moderate problems, and severe problems. To standardize the data, utility was estimated using Taiwan population weights [26]. The utility value ranged from 0 to 1, where 0 indicated death and 1 indicated perfect health. Notably, negative values indicated health statuses considered worse than death.

### Statistical analysis

Descriptive statistics were used to summarize the baseline characteristics of the study cohort. Continuous variables are described using the mean with the standardized deviation (SD) or the median with 25th and 75th percentiles. Categorical variables are described using frequencies and proportions. The features (or predictors) included age, sex, marital status, family support, household income, education level, BMI, the number of medications used, CCI, physical illness, cognitive impairment, depression, pain, urinary incontinence, falls, activities of daily living, frailty, social and emotional loneliness, and malnutrition. Two variables, metastatic solid tumor and human immunodeficiency virus/acquired immunodeficiency syndrome, were excluded from the analysis due to a 0% prevalence rate. In total, thirty-six features were included for modeling. The target variable was utility, which was converted using the EQ-5D scale.

### Multiple imputation

Missing values appear in the BMI and CCI variables, and their percentages of missing was 2.5% and 0.6%. To reduce the bias of missing, we used multiple imputation by chained equations (MICE) to impute missing data [27, 28]. MICE is a multiple imputation technique that is a robust and informative method to deal with missing data in datasets [29]. We assumed that given the features used in the imputation procedure were missing at random. The MICE procedure runs a series of regression models in which each feature with missing data is modeled conditional on the other variables in the data set. This process continues until all specified variables have been imputed.

### Choosing a machine learning algorithm

The random forest algorithm was employed to assess feature importance in predicting utility. This algorithm was selected for its utilization of bootstrap aggregation (bagging), an ensemble meta-algorithm that enhances model stability, accuracy, and robustness [30]. Hyperparameters, including the number of features for potential node splitting, tree quantity, and maximum tree depth, were optimized through sensitivity analyses to maximize predictive performance. Results are presented in supplementary Figs. 1 and 2.

### Model performance

Model performance was evaluated using 5-fold cross-validation, a resampling procedure designed for limited data samples. The dataset was randomly partitioned into five subsets, with each subset serving as test data once while the remainder functioned as training data. This process was iterated five times to mitigate variability. Performance metrics, including R-squared (R^2^), mean square error (MSE), and mean absolute error (MAE), were averaged across iterations to estimate the model’s predictive capacity on novel data. Superior model performance was indicated by larger R^2^ values and smaller MSE and MAE values.

### Machine learning interpretation: ranking variable importance, accumulated local effects, and feature interaction

Despite its power and robustness, the random forest algorithm lacks intuitive interpretability due to its black-box nature. To address this limitation, model-agnostic methods were employed to elucidate feature-target relationships:

1. Permutation feature importance: Quantifies the increase in prediction error after feature value permutation, with higher errors indicating greater importance [31].
2. Accumulated local effects (ALE) plots: Illustrate average feature effects on model predictions [32].
3. Feature interactions: Friedman’s H-statistic measures interaction strength, with values > 0.1 considered significant [33, 34].

These methods bridge the gap between random forest’s robust, personalized forecasting and the interpretability of traditional regression tools, which provide clear coefficient interpretations for average effects, strength, and directionality. Analysis focused on two-way interactions among the top three most important features. Implementations utilized R (version 4.2.0) and Quanta for Medical Care AI: AI Medical Platform (QOCA aim) v. 2.4.9 (Quanta Computer Inc., Taoyuan, Taiwan), with the R-package ranger for random forest and R-package interpretable machine learning (iml) for ALE and interaction plots.

## Results

From June to September 2018, there were 283 adults aged ≥ 65 years admitted to medical wards. After excluding those with critical or terminal illness (*n* = 3), those with hospital stay < 72 h (*n* = 2), and those with impaired communication abilities (*n* = 46), remaining 232 participants. Of 232 individuals, 72 refused to participate the study, resulting in a total of 160 older adults included in the final study cohort (Supplementary Fig. 3). The mean age was 79 years (SD 8 years). There were 52 individuals (32%) aged 65 to 74 years, 64 individuals (40%) aged 75 to 84 years, and 44 individuals (28%) aged ≧ 85 years. The proportion of males (55%) was higher than that of females (45%). The older adults who were married or cohabiting was 34%. 93% of the older adults were satisfied with their family support. 11% of the older adults identified their household income as wealthy or well-off. 41% of the older adults had a high school education. 62% of the older adults had a BMI in the range of 18–24 kg/m^2^. The five most common physical comorbidities were renal disease (28%), any malignancy (19%), cerebrovascular disease (18%), congestive heart failure (14%), and peptic ulcer (12%). 49% of the older adults had a pain problem, and 41% had a history of falls. The average ADL scale was 7 (SD 10), and the average clinical frailty scale was 3.5 (SD 1.8) (Table 1).

**Table 1:** The baseline characteristics of the study population

Variable	*N* = 160
Age, mean (standardized deviation)	79 (8)
Age group, n (%)	
65–74 years	52 (32%)
75–84 years	64 (40%)
85 + years	44 (28%)
Sex, n (%)	
Female	72 (45%)
Male	88 (55%)
Marriage	55 (34%)
Satisfaction with family support	149 (93%)
Household income: wealthy or well-off, n (%)	17 (11%)
Level of education, n (%)	
Illiterate	33 (21)
Elementary school	62 (39)
High school or above	65 (41)
Body mass index, mean (standardized deviation)	23.5 (4.0)
Body mass index, n (%)	
< 18 kg/m^2^	10 (6%)
18–24 kg/m^2^	99 (62%)
25 + kg/m^2^	51 (32%)
Number of medicines used, mean (standardized deviation)	3.2 (3.5)
Number of medicines used, n (%)	
0–1	48 (30%)
2–4	79 (49%)
5+	33 (21%)
Charlson comorbidity index, mean (standardized deviation)	2.3 (1.9)
Charlson comorbidity index, n (%)	
0	33 (21%)
1–2	63 (39%)
3–4	42 (26%)
5+	22 (14%)
Comorbidity, n (%)	
Diabetes without chronic complications	48 (30%)
Renal disease	44 (28%)
Any malignancy	31 (19%)
Cerebrovascular disease	28 (18%)
Congestive heart failure	23 (14%)
Peptic ulcer	19 (12%)
Chronic pulmonary disease	18 (11%)
Mild liver disease	13 (8.1%)
Diabetes with chronic complications	13 (8.1%)
Myocardial infarction	12 (7.5%)
Dementia	11 (6.9%)
Rheumatic disease	5 (3.1%)
Hemiplegia or paraplegia	3 (1.9%)
Peripheral vascular disease	2 (1.2%)
Moderate to severe liver disease	1 (0.6%)
Metastatic Solid Tumor	0 (0%)
Human immunodeficiency virus/Acquired immunodeficiency syndrome	0 (0%)
Cognitive impairment, n (%)	31 (19%)
Depression, n (%)	56 (35%)
Pain, n (%)	79 (49%)
Urinary incontinence, n (%)	37 (23%)
Falls, n (%)	65 (41%)
Activities of daily living scale, mean (standardized deviation)	7 (10)
Activities of daily living scale, median (p25, p75)	0 (0, 10.3)
Clinical frailty scale, mean (standardized deviation)	3.5 (1.8)
Clinical frailty scale, median (p25, p75)	3 (2, 5)
Social loneliness, mean (standardized deviation)	1.5 (2)
Social loneliness, median (p25, p75)	0 (0, 4)
Emotional loneliness, mean (standardized deviation)	1.2 (1.5)
Emotional loneliness, median (p25, p75)	1 (0, 2)
Malnutrition universal screening tool, mean (standardized deviation)	0.43 (0.9)
Malnutrition universal screening tool, median (p25, p75)	0 (0, 0)
Quality of life, mean (standardized deviation)	0.74 (0.26)
Quality of life, median (p25, p75)	0.8 (0.6, 1.0)

The predictive performance of the random forest model was evaluated, with an average R^2^ value of 0.73, MSE of 0.018, and MAE of 0.093 obtained from 5-fold cross-validation. The R^2^ is closer to 1 which indicates a better fit of the model. Lower MSE and MAE values indicate a model with better predictive accuracy, as it signifies that the predicted values are close to the actual values. Notably, during each round of cross-validation, little variance was observed for R^2^, MSE, and MAE when compared to the average values. This indicated that the predictions of the random forest model were robust and reliable (Supplementary Table 1).

**Fig. 1:**
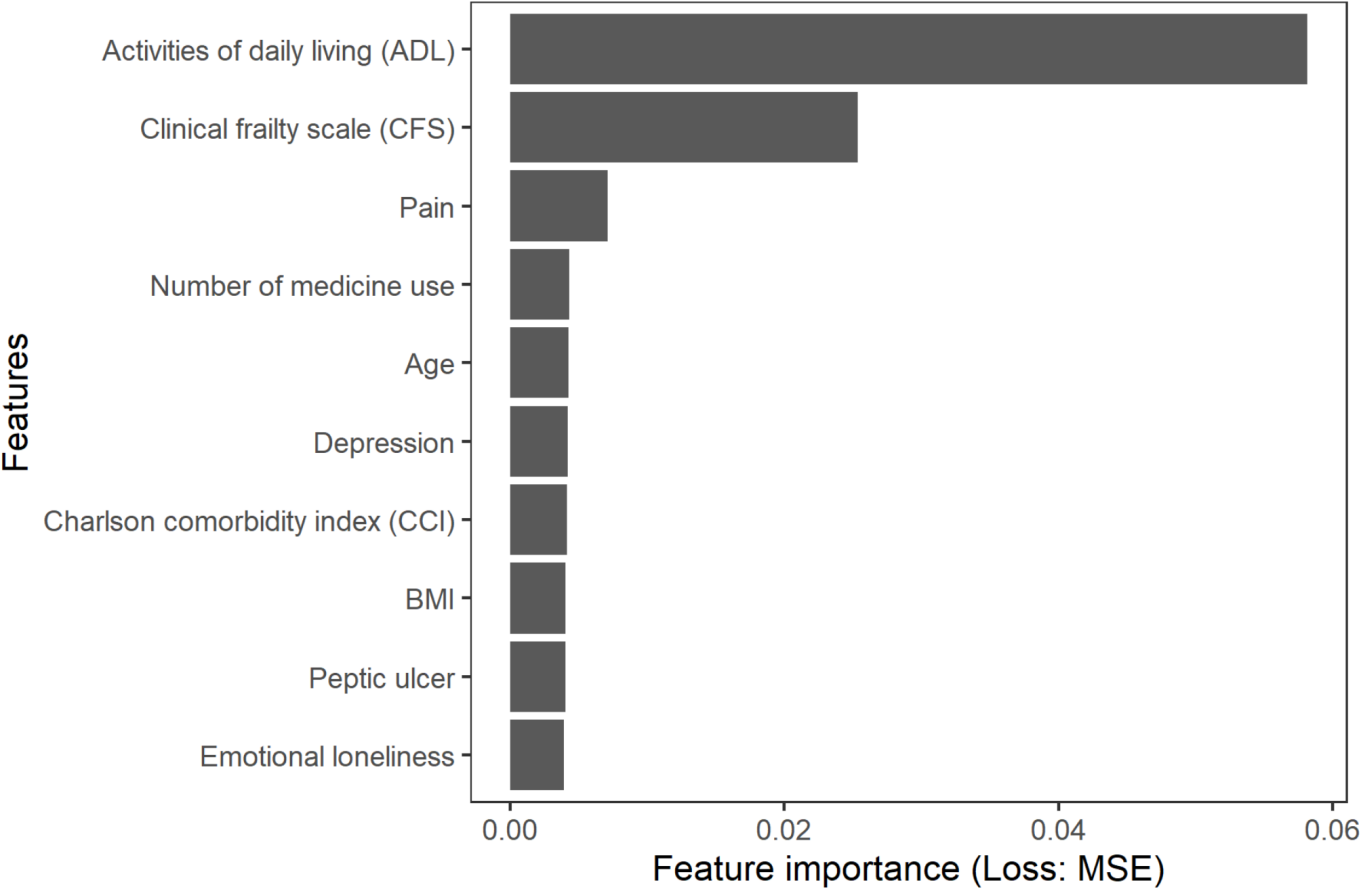
Permutation feature importance of the top 10 features for quality of life prediction. *Abbreviations*: ADL: activities of daily living; CCI: Charlson Comorbidity Index, CFS: clinical frailty scale; MSE: mean square error of estimation

The top predictors of utility, in descending order of importance, were ADL, frailty, pain, medication count, age, depression, CCI, BMI, peptic ulcer, and emotional loneliness. ADL, frailty, and pain exhibited the most significant influence on utility prediction (Fig. 1).

**Fig. 2:**
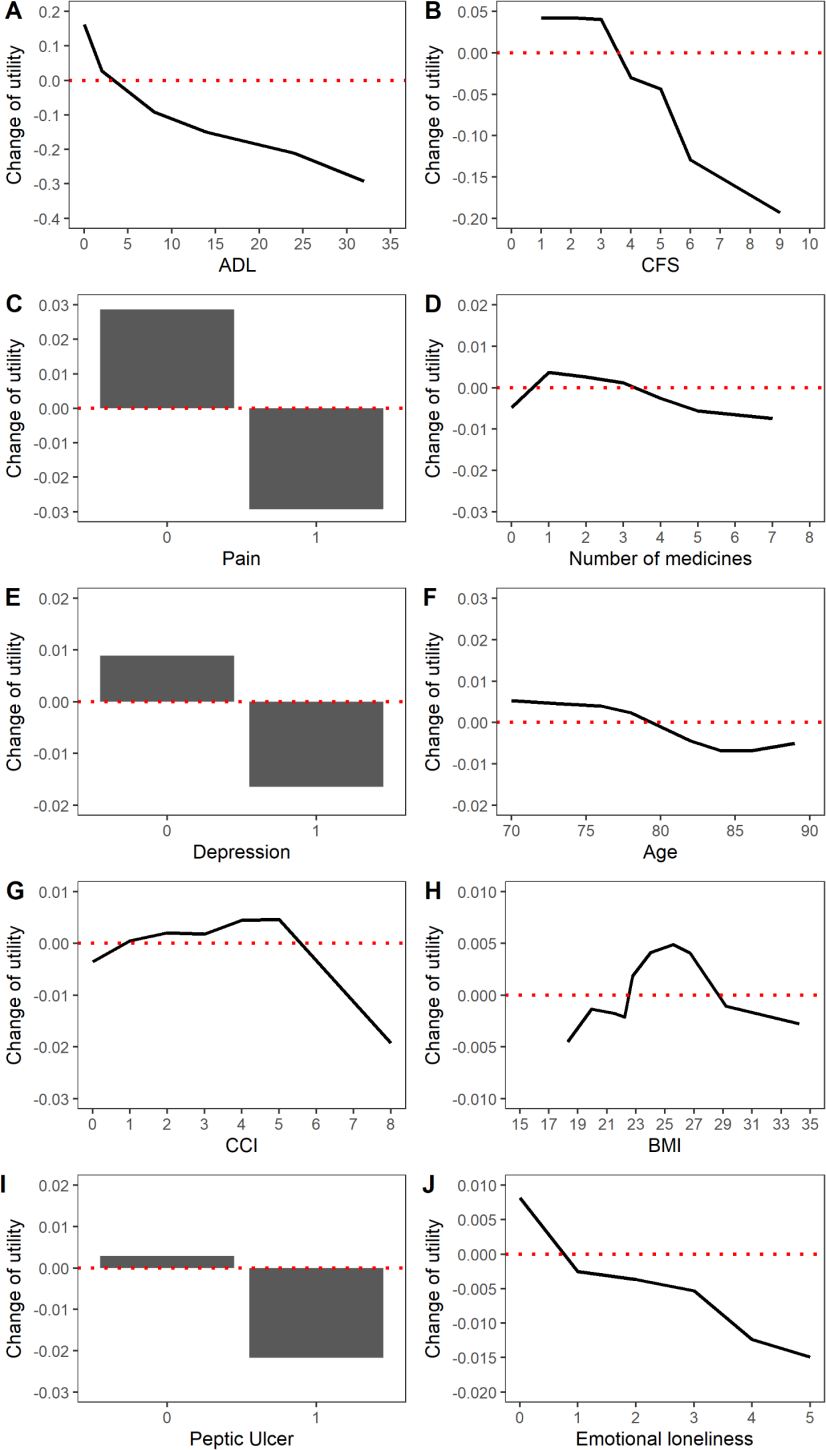
Accumulated local effects for the predicted change in quality of life of the prediction model with changes in features. *Abbreviations*: ADL: activities of daily living; BMI: body mass index, CCI: Charlson Comorbidity Index, CFS: clinical frailty scale

The ALE plot analysis revealed that CFS > 4 was associated with decreased utility, which aligns with the expert-based decision logic. A non-linear relationship was observed between BMI and utility, with values less than 20 kg/m² or greater than 28 kg/m² correlating with reduced QOL. ADL and emotional loneliness demonstrated negative linear correlations with utility. The presence of pain, depression, or peptic ulcer was associated with average QOL decreases of 0.03, 0.015, and 0.02, respectively (Fig. 2).

**Fig. 3:**
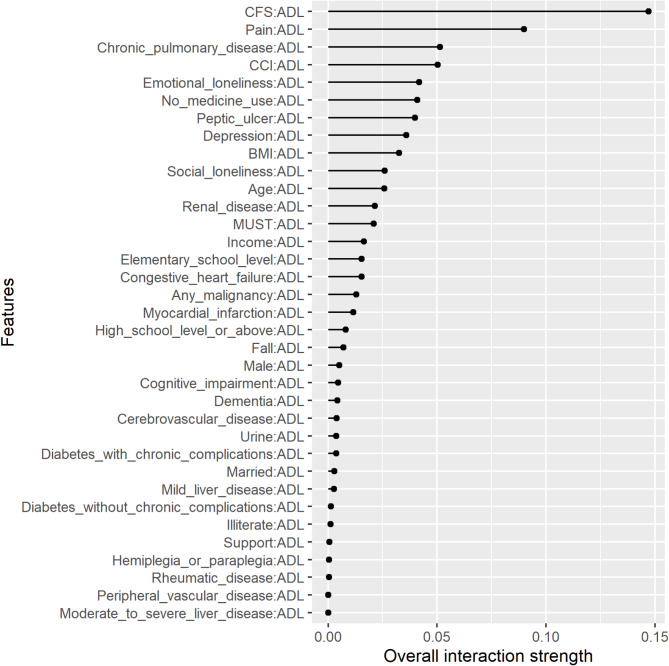
Two-way interactions of the feature of activities of daily living with other features in the prediction model. *Abbreviations*: ADL: activities of daily living; BMI: body mass index, CCI: Charlson Comorbidity Index, CFS: clinical frailty scale, MUST: malnutrition universal screening tool

**Fig. 4:**
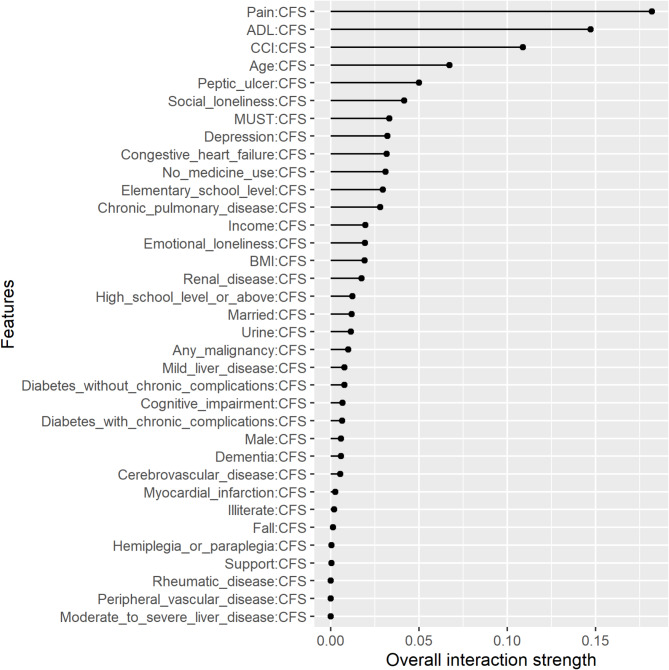
Two-way interactions of the feature of frailty with other features in the prediction model. *Abbreviations*: ADL: activities of daily living; BMI: body mass index, CCI: Charlson Comorbidity Index, CFS: clinical frailty scale, MUST: malnutrition universal screening tool

**Fig. 5:**
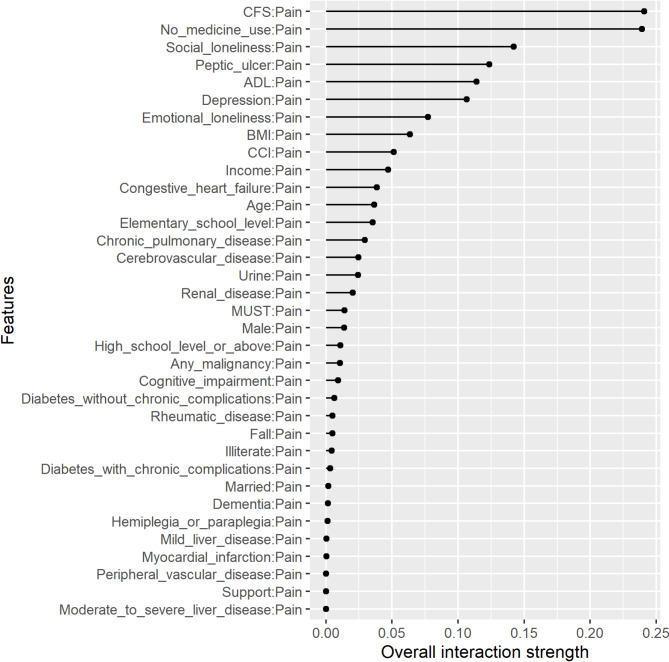
Two-way interactions of the feature of pain with other features in the prediction model. *Abbreviations*: ADL: activities of daily living; BMI: body mass index, CCI: Charlson Comorbidity Index, CFS: clinical frailty scale, MUST: malnutrition universal screening tool

Figures 3, 4 and 5 show the two-way interactions for ADL, frailty, and pain, each interacting with other characteristics. Regarding ADL, 14.7% of the effect of ADL came from its interaction with CFS. Frailty significantly interacted with pain and ADL. 18% of the effect of frailty came from its interaction with pain. Pain significantly interacted with frailty, the number of medications used, social loneliness, peptic ulcer, ADL, and depression. Among these features, pain and frailty had significantly higher strengths of interaction.

## Discussion

We demonstrated that ADL, frailty and pain were significantly associated with the prediction of QOL. In light of previous reports, one study showed frailty and depression were associated with poor QOL in older adults receiving home-based health care [35], and the other two studies revealed a negative relationship between frailty and QOL [36, 37]. The discrepancies between our findings and those of previous studies may be attributed to several factors. First, the previous studies included community-dwelling older adults [35–37], whereas the current study population comprised hospitalized older adults. Second, the measurement tools differed, including the World Health Organization Quality of Life-BREF by Tasioudi et al. [35] and Papathanasiou et al. [37], principal component analysis by Goyal & Mohanty [36], and EQ-5D in the current study.

In the current study, machine learning indicated that frailty with a CFS ≥ 4 was associated with worse quality of life (QOL) (Fig. 2B). It is important to note that CFS ≥ 4 is relevant to the clinical definition of living with frailty [23]. Frailty significantly diminishes muscle strength and endurance in older adults, leading to functional disability, and further compromises their QOL [38].

We also showed an inverted U-shaped relationship between BMI and QOL using machine learning, which is consistent with previous studies [39–42]. In addition, the current study showed that BMI of approximately 25–26 kg/m^2^ was associated with the best QOL. A previous study [41] reported that the best QOL was observed among males with BMI of approximately 22–29 kg/m^2^ and among females with BMI of approximately 22 kg/m^2^. Another study [39] proposed that an increase in the change of BMI by 2.50 kg/m^2^ in females (median was 26.10 kg/m^2^) and 5.19 kg/m^2^ in individuals with at least one disease was associated with the highest QOL. Our findings were consistent with those of previous studies that employed a machine learning model. Individuals with obesity or underweight may experience adverse effects on social and mental domains, which can result in negative self-perception and social interactions, ultimately leading to a decline in their QOL [39].

We demonstrated pain was associated with poor QOL, compatible with previous report [36]. Individuals aged 65 and above who experience pain tend to report lower QOL, which can be attributed severe health and environmental challenges [43]. The management of pain in older adults requires special attention due to the presence of various physiological, cognitive, functional, and social factors that may undergo change with advancing age, necessitating a tailored approach [44].

We revealed depression was associated with poor QOL, consistent with previous report [35]. Depression in older adults is not a normal part of aging process [45]. Late-life depression is distinguished by an atypical presentation of symptoms, including a greater prevalence of somatic symptoms relative to mood symptoms, rendering the condition challenging to detect and treat [46]. Nevertheless, older adults who are aware of their health status may have the potential to enhance their QOL [47].

In addition to the association between multiple geriatric syndromes and QOL, we further investigated the effect of the interactions among multiple geriatric syndromes on QOL in older adults. Our results indicated that 14.7% of the effect of ADL on QOL came from its interaction with frailty (Fig. 3), and 18% of the effect of frailty on QOL came from its interaction with pain (Fig. 4). Previous studies have reported that geriatric syndromes may interact with each other, chronic diseases or medications used; for example, one study reported that the interactions among geriatric syndromes predicted 3-month mortality [48], another study investigated the interactions between geriatric syndromes and chronic diseases [9], and another study showed the interaction between drugs and geriatric syndromes [49].

The ALE plot, a model-agnostic method, elucidates feature impacts on predictions in complex machine learning models. It operates by segmenting feature values and examining localized prediction changes, subsequently accumulating these effects to demonstrate overall feature influence. In ALE plots, the X-axis represents feature values, while the Y-axis depicts accumulated effects, with positive values indicating increased prediction and negative values indicating decreased prediction. For slope characteristics, steepness indicates effect strength, direction reveals effect nature, flatness suggests minimal impact, and non-linearity (e.g., U-shape) implies complex relationships. This approach enhances model transparency, facilitating comprehensive understanding of feature effects and supporting informed decision-making.

Friedman’s H-statistic quantifies feature interaction effects on prediction variance. It ranges from 0 (no interaction) to 1 (all variance explained by partial dependence function sums). Interactions with H > 0.1 are considered significant, with the magnitude indicating the proportion of a feature’s effect attributable to its interaction with another feature.

The current study has several strengths. First, to our knowledge, it is the first study to demonstrate the effects of interactions among multiple geriatric syndromes on QOL. Second, machine learning can deal with the high-dimensional exploration of complex data and can identify nonlinear and interactive effects [50]. When the underlying mechanism of a research question is complex, machine learning-based methodologies can be used to investigate the associations among the factors of interest. Third, machine learning models’ capacity to capture complex interactions among geriatric syndromes offers insights for personalized interventions. For example, strong frailty-pain interaction in QOL prediction suggests a need for integrated clinical approaches, while non-linear BMI-QOL relationship underscores the importance of individualized weight management strategies for older adults. Despite the incomplete understanding of the relationship between frailty and pain, recent evidence [51] has indicated the presence of a bidirectional relationship between the two. This relationship has the potential to create a vicious cycle, whereby each serves to accelerate the progression of the other [51]. It underscores the importance of pain assessment and management in the recognition and prevention of frailty [52]. Integration of these algorithms into clinical workflows could facilitate risk stratification based on unique combinations of geriatric syndromes, enabling timely, targeted interventions.

The study has inherent limitations. First, this was a single-center study with a small sample size. This could have led to variability in the data, which we addressed by using cross-validation to assess model performance. Second, we recruited only older adults hospitalized with medical conditions, so the results may not be generalizable to community-dwelling older adults. Third, the number of medications used was self-reported or proxy-reported, which may have introduced recall bias. Fourth, although the random forest algorithm offers robustness and excellent predictive performance, it may be susceptible to bias when dealing with categorical features with multiple levels, leading to an overemphasis on the importance of these features. However, all categorical features were binary in the current study, which would reduce the bias. Fifth, the overlap in domains of EQ-5D may lead to overestimation of relationships, and difficulty in isolating unique contributions to QOL. Ultimately, given the current study’s cross-sectional design and lack of environmental factors, further research is necessary to elucidate the longitudinal trajectories of geriatric syndromes and environmental factors with their impact on QOL over time.

## Conclusion

The machine-learning model was able to identify a number of factors that were important in the prediction of QOL, including ADL, frailty, pain, the number of medications used, age, depression, CCI, BMI, peptic ulcer, and emotional loneliness. Among these factors, ADL, frailty, and pain significantly interacted with the other risk factors. These interactions suggest that ADL, frailty, and pain have complex effects on QOL in older adults. These interactions need to be considered in clinical decision-making.

## Electronic supplementary material

Below is the link to the electronic supplementary material.

Supplementary Material 1

## Data Availability

No datasets were generated or analysed during the current study.

## Abbreviations

ADL: Activities of daily living
ALE: Accumulated local effects
BMI: Body mass index
CCI: Charlson Comorbidity Index
CFS: clinical frailty scale
CGA: Comprehensive geriatric assessment
EQ-5D-3L: Three-level five-dimensional Euro-Quality of Life tool
GDS-5: Geriatric depression scale-5
MAE: Mean absolute error of estimation
MICE: Multiple imputation by chained equations
MSE: Mean square error of estimation
MUST: Malnutrition universal screening tool
QOL: Quality of life
SD: Standardized deviation
