# Supplementary material for: Predictive model to identify multiple synergistic effects of geriatric syndromes on quality of life in older adults: a hospital-based pilot study
Source: BMC Geriatr. 2025 Apr 26;25:283. doi: 10.1186/s12877-025-05931-8 (PMC12032690; doi:10.1186/s12877-025-05931-8)
Supplement: Supplementary file 1 — Supplementary Material 1 [file 12877_2025_5931_MOESM1_ESM.docx]

Supplementary Table 1. Prediction performance metrics for the prediction model with 5-fold cross validation

| K-fold | R^2^ | Mean square error of estimation | Mean absolute error of estimation |
| --- | --- | --- | --- |
| 1 | 0.723 | 0.015 | 0.089 |
| 2 | 0.805 | 0.041 | 0.126 |
| 2 | 0.724 | 0.015 | 0.093 |
| 4 | 0.695 | 0.008 | 0.072 |
| 5 | 0.711 | 0.010 | 0.083 |
| Mean | 0.732 | 0.018 | 0.093 |

Supplementary Figure 1. Sensitivity to the performance of the mean square error of estimation in different numbers of trees


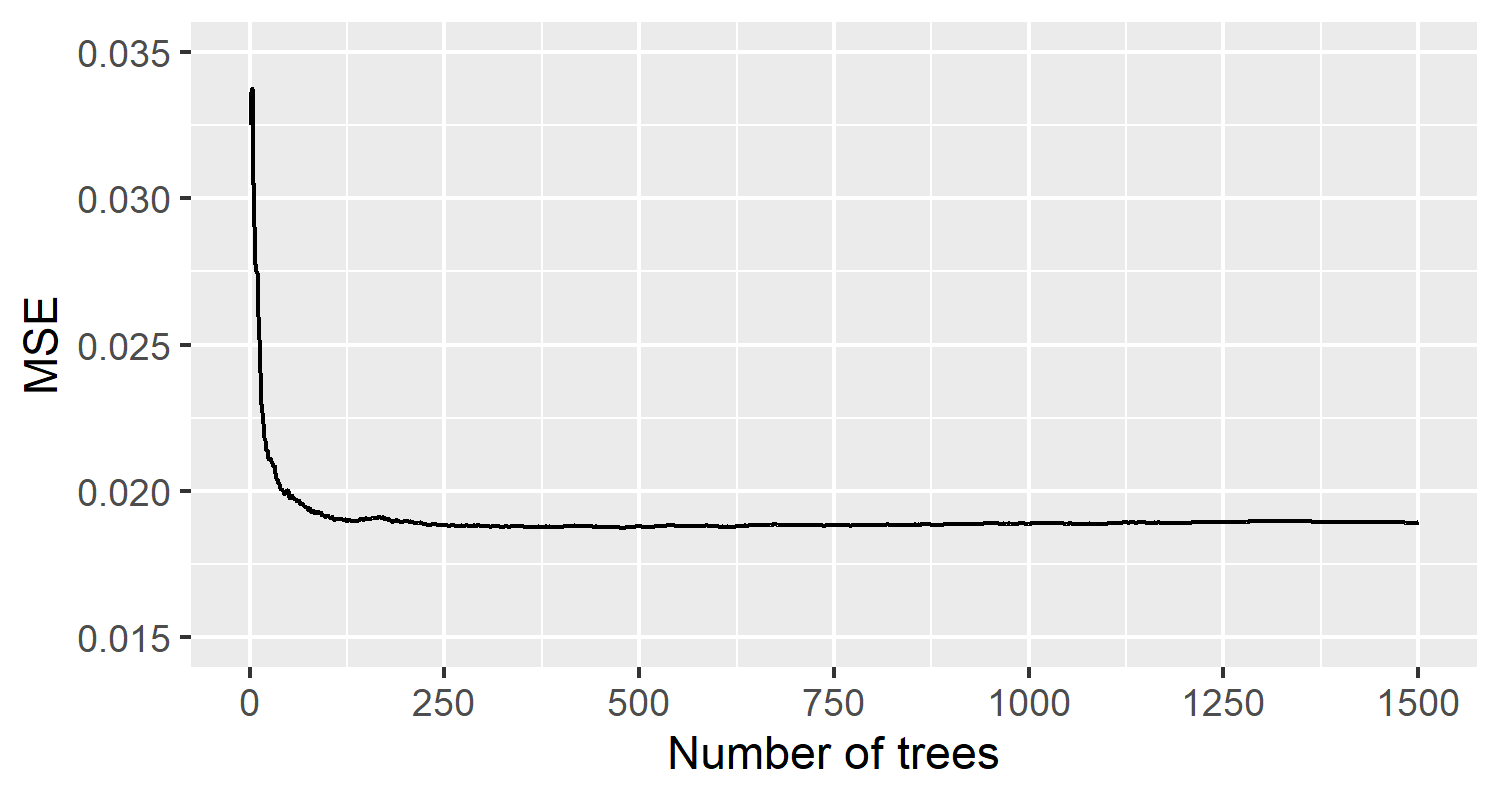


*Note*: When the number of trees reaches 250, the mean square error of estimation (MSE) tends to gradually stabilize, and the MSE is maintained at approximately 0.018.

Supplementary Figure 2. Sensitivity to the performance of the R^2^ value of estimation at different depths of trees


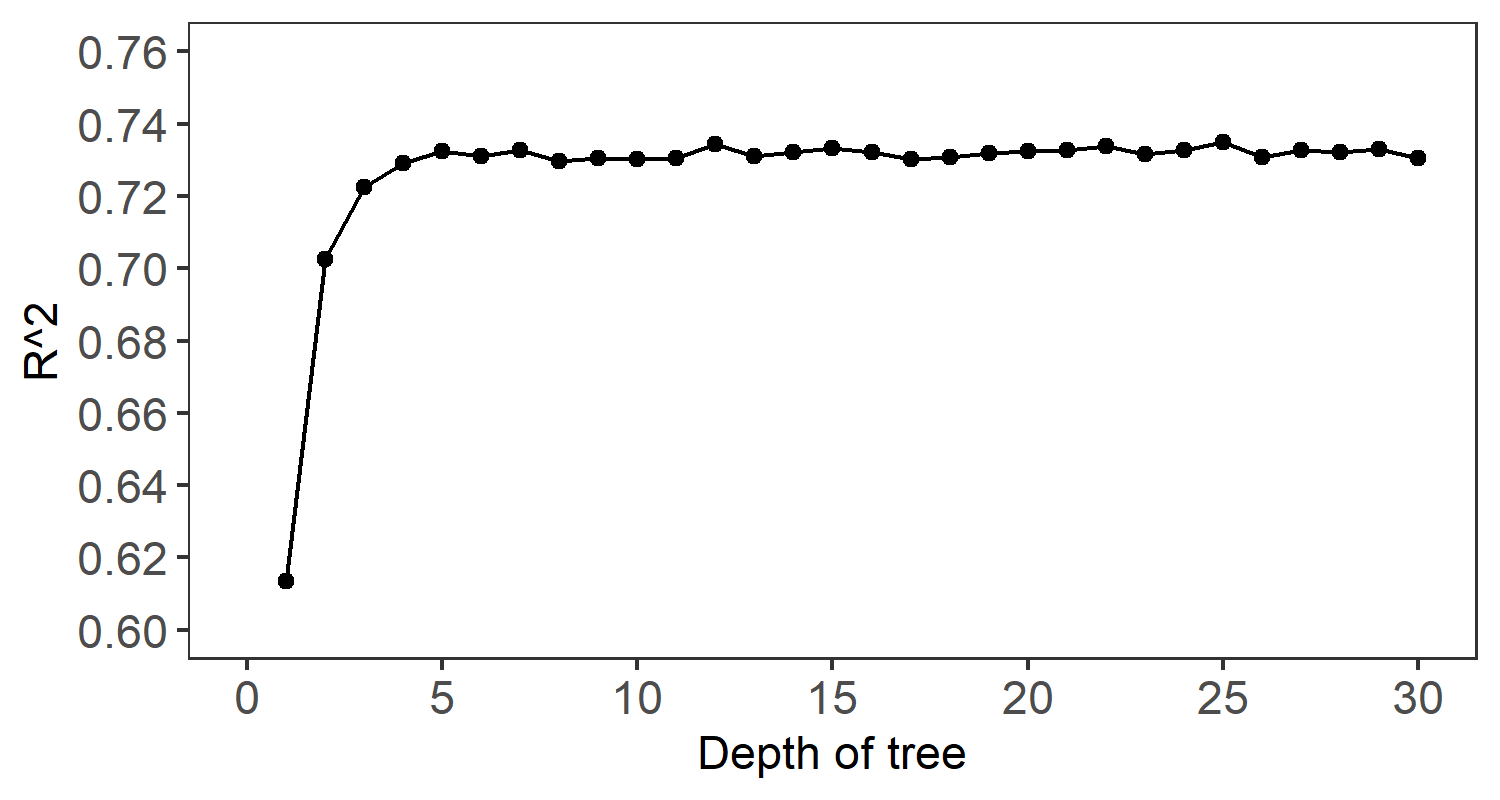


*Note*: The R^2^ value stabilizes and remains at approximately 0.73 when the depth of the trees reaches 5.

Supplementary Figure 3. Flowchart of the participants selection

All participants (n=283)

1. Excluding critical or terminal illness (n=3)
2. Excluding hospital stay<72 hours (n=2)
3. Excluding those with impaired communication abilities (n=46)

Remaining participants (n=232)

Refuse to participate (n=72)

Final participants (n=160)
